# Update on Preclinical Development and Clinical Translation of Cholecystokinin-2 Receptor Targeting Radiopharmaceuticals

**DOI:** 10.3390/cancers13225776

**Published:** 2021-11-18

**Authors:** Elisabeth von Guggenberg, Petra Kolenc, Christof Rottenburger, Renata Mikołajczak, Alicja Hubalewska-Dydejczyk

**Affiliations:** 1 Department of Nuclear Medicine, Medical University of Innsbruck, 6020 Innsbruck, Austria; 2 Department of Nuclear Medicine, University Medical Centre Ljubljana, 1000 Ljubljana, Slovenia; petra.kolenc@kclj.si; 3 Faculty of Pharmacy, University of Ljubljana, 1000 Ljubljana, Slovenia; 4 Division of Nuclear Medicine, University Hospital Basel, 4031 Basel, Switzerland; christof.rottenburger@usb.ch; 5 National Centre for Nuclear Research, Radioisotope Centre POLATOM, 05-400 Otwock-Świerk, Poland; renata.mikolajczak@polatom.pl; 6 Chair and Department of Endocrinology, Jagiellonian University Medical College, 30-688 Cracow, Poland; alahub@cm-uj.krakow.pl

**Keywords:** cholecystokinin-2 receptor, agonist, antagonist, tumor targeting, molecular imaging, targeted radiotherapy, medullary thyroid carcinoma, radiopharmaceuticals

## Abstract

**Simple Summary:**

Peptide analogs, derived from the natural peptide hormone gastrin, are promising candidates for improving the visualization and treatment of tumors. Gastrin specifically binds to the cholecystokinin-2 receptor, a G-protein-coupled receptor expressed on the cell surface of different tumors. This enables specific targeting of tumor cells using gastrin analogs, labeled with radioisotopes. The receptor is expressed at high incidence in medullary thyroid carcinoma, a rare form of thyroid cancer lacking effective treatments at an advanced stage. Different radiolabeled gastrin analogs as well as nonpeptidic compounds targeting CCK2R have been developed. Specific modifications have been introduced in order to safely deliver the radiation to the tumor site. In this review, recent strategies applied to improve the targeting properties are described. These developments enabled the introduction of new radiolabeled peptide analogs for imaging and therapy in cancer patients. In addition to highlighting the current clinical trials, the perspectives for future applications are given.

**Abstract:**

The cholecystokinin-2 receptor (CCK2R) has been a target of interest for molecular imaging and targeted radionuclide therapy for two decades. However, so far CCK2R targeted imaging and therapy has not been introduced in clinical practice. Within this review the recent radiopharmaceutical development of CCK2R targeting compounds and the ongoing clinical trials are presented. Currently, new gastrin derivatives as well as nonpeptidic substances are being developed to improve the properties for clinical use. A team of specialists from the field of radiopharmacy and nuclear medicine reviewed the available literature and summarized their own experiences in the development and clinical testing of CCK2R targeting radiopharmaceuticals. The recent clinical trials with novel radiolabeled minigastrin analogs demonstrate the potential for both applications, imaging as well as targeted radiotherapy, and reinforce the clinical applicability within a theranostic concept. The intense efforts in optimizing CCK2R targeting radiopharmaceuticals has led to new substances for clinical use, as shown in first imaging studies in patients with advanced medullary thyroid cancer. The first clinical results suggest that the wider clinical implication of CCK2R-targeted radiopharmaceuticals is reasonable.

## 1. Introduction

The cholecystokinin-2 receptor (CCK2R) is a promising target for theranostic use in nuclear medicine, and has been in the focus of the radiopharmaceutical development over the last twenty years. The expression of this receptor at high incidence and density has been proven mainly for medullary thyroid carcinoma (MTC) and small cell lung cancer (SCLC) [1]. Furthermore, CCK2R expression has been confirmed for gastrointestinal stromal tumors, astrocytomas and stromal ovarian cancers [2]. In addition, CCK2R targeting might be of additive value for gastroenteropancreatic and bronchopulmonary neuroendocrine tumors, especially insulinomas, vipomas, as well as bronchial and ileal carcinoids [3].

The development of radiolabeled CCK2R targeting peptide analogs was initiated in the late 1990s. Several pioneers laid the foundation for this new diagnostic and therapeutic approach. Strong CCK2R expression, especially in MTC and its metastases, was demonstrated by the group of Prof. Jean Claude Reubi [4]. First scintigraphic visualization of tumor lesions in a patient with metastasised MTC using ^131^I-labeled gastrin was undertaken by Thomas Behr in 1998 [5]. In this team, Martin Béhé developed the first ^111^In-labeled gastrin derivatives with selective affinity for CCK2R [5,6]. The research group around Marion de Jong worked on non-sulphated cholecystokinin analogs. By increasing specificity for CCK2R over CCK1R, reduced uptake in normal tissue expressing CCK1R was achieved [7]. Soon thereafter, additional research groups across Europe directed their attention to the preclinical development of peptide-based CCK2R targeting probes.

Since the very beginning, the difficulties in the development of clinically useful radiolabeled CCK2R targeting peptide analogs became clear. These were related either to high kidney uptake, leading to nephrotoxicity during therapeutic application, or low enzymatic stability, limiting the tumor targeting properties [8,9]. The clinical comparison of three different ^111^In- and ^99m^Tc-labeled derivatives of human minigastrin (MG) and cholecystokinin-8 demonstrated the need for further radiopharmaceutical development to enable CCK2R-based peptide receptor radionuclide therapy (PRRT) [10]. A small, but well networked radiopharmaceutical community accelerated the radiopharmaceutical development. Within the COST (European Cooperation in Science and Technology) Action BM0607 on Targeted Radionuclide Therapy, twelve different CCK2R targeting peptide analogs were preclinically evaluated, with the major aim of finding a promising new candidate for PRRT [11,12,13]. These joint efforts triggered further clinical studies and gave new directions to the ongoing preclinical research.

This review highlights the recent clinical achievements and new advances in the development of CCK2R targeting radiopharmaceuticals. Most of the clinical experience available has been gained for patients with advanced MTC. Therefore, the clinical challenges in the treatment of this specific tumor are discussed and the presentation of the diagnostic and therapeutic potential of CCK2R targeting peptide analogs is focused on this patient group. In addition, perspectives on the application of this new group of radiopharmaceuticals in patients with CCK2R expressing tumors and on the goals to be pursued in future clinical studies are given.

## 2. Current Radiopharmaceutical Development

Various structural modifications of CCK2R targeting peptide analogs have been investigated in the past. These were mainly aimed at increasing the metabolic stability of the linear peptide sequence and improving the overall biodistribution profile of the radiolabeled analogs. The modifications included the depletion of the penta-Glu sequence, the introduction of D-amino acids, and the substitution of oxidation-sensitive methionine, cyclization and multimerization [11,12,14,15,16,17,18]. These developments resulted in the clinical translation of two MG analogs, namely [^111^In]In-CP04 (PP-F11) and [^177^Lu]Lu-PP F11N (for amino acid sequence see Table 1). Both radiopeptides are currently being evaluated in phase I clinical trials [19,20].

**Figure 1:**
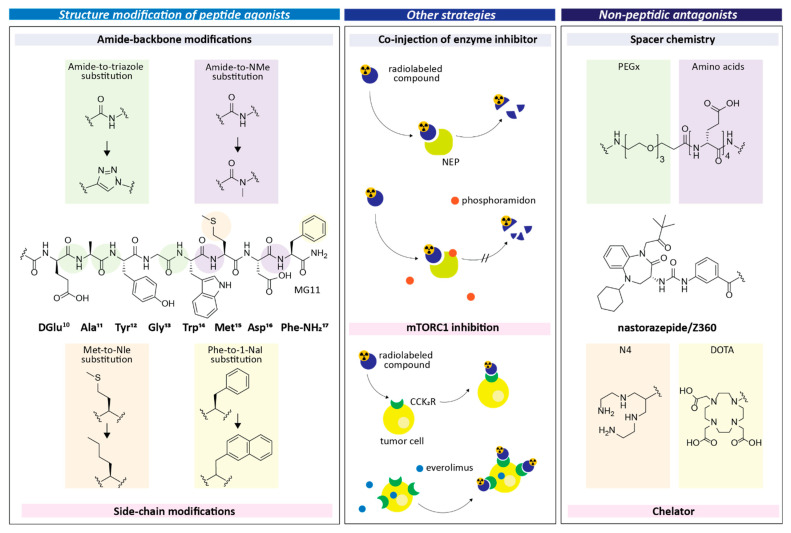
Recent developments aiming towards optimizing CCK2R targeting (as described in references: [21,22,23,24,25,26,27,28,29,30,31,32,33,34,35,36]).

However, the preclinical development and search for optimal CCK2R targeting probes is still ongoing. A major issue remains metabolic stability, possibly affecting the tumor uptake and retention. Therefore, various attempts for further optimization of the peptide sequence are pursued currently. Other strategies to improve tumor uptake are directed towards enhancing the CCK2R expression or increasing the radiation dose at the tumor sites. Furthermore, nonpeptidic compounds have been developed recently in an attempt to improve tumor uptake and retention in combination with low kidney retention. These developments are summarized in Figure 1 and described more in detail in the following section.

The application of enzyme inhibitors was a pursued strategy to preserve radiopeptides from metabolic degradation and thereby increase their availability for specific receptor binding [25]. An increased percentage of intact radiopeptide was detected in the blood circulation of mice after co-injection of various radiolabeled MG analogs together with the neutral endopeptidase (NEP) inhibitor phosphoramidon (PA) [25]. Increased in-vivo metabolic stability resulted in improved radiopeptide uptake in CCK2R-expressing tumor xenografts in mice, as shown in Table 1, for selected peptide analogs.

The highest impact of PA inhibition was observed for the highly unstable radiopeptide [^111^In]In-DOTA-MG11, for which impaired tumor uptake due to its very low metabolic stability has been shown previously [21,22,26,37]. Co-injection of PA was tested in SCID mice bearing xenografts of AR42J cells expressing rat CCK2R, as well as stably transfected A431 cells expressing the human CCK2R. The tumor uptake of [^111^In]In-DOTA-MG11 was increased by a factor of eight (from less than 2%ID/g to more than 16%ID/g, at 4 h after injection), while kidney retention remained at low levels [21,25]. NEP inhibition influenced the tumor uptake of metabolically stable analogs such as [^111^In]In-DOTA-MG0 or [^111^In]In-CP04, to a slightly lesser extent [22]. The authors suggested that the concept of enzyme inhibition might be a rational alternative to costly and time-consuming development of compound libraries. On the other hand, the tumor targeting properties of [^177^Lu]Lu-PP-F11N alone were comparable to [^177^Lu]Lu-DOTA-MG11 in the presence of NEP inhibitors, pointing out that the human application of a single compound might be more straightforward and therefore desirable [23].

Since co-injection of the NEP inhibitor PA had a significant effect on metabolic stability and consequently the tumor uptake of [^111^In]In-DOTA-MG11, several research groups have focused on site-specific stabilization of MG11 to circumvent its fast degradation by proteases. Oxidation sensitive Met was replaced by Nle to avoid loss of receptor affinity [37]. The strategy of amide-to-triazole substitution was investigated for DOTA-[Nle^6^]MG11 and PP-F11N [27,28,29]. Single insertion of 1,4-disubstituted 1,2,3-triazole as a bioisostere of the trans-amide bond between Tyr^3^ and Gly^4^ led to improved receptor affinity. Using a computational model of the ligand bound to CCK2R, a contribution of the aromatic triazole heterocycle to the binding affinity was suggested [27]. For peptide derivatives with multiple 1,2,3-triazoles, an additional effect improving the metabolic stability was found for amide-to-triazole substitution between DGlu^1^ and Ala^2^. Even a derivative with three consecutive substitutions retained receptor affinity and showed improved tumor targeting over [^177^Lu]Lu-DOTA-[Nle^6^]MG11 [29]. As shown in Table 2, stabilization of the peptide backbone by triazolo substitution yields a similar improvement of the tumor targeting properties as co-injection of PA [22,29].

In further studies on the structure-activity relationship performed with CP04 (PP-F11), a possible role of stabilizing ionic interactions of negatively charged amino acids with a positively charged extracellular domain of the receptor was described, resulting in improved performance of radiolabeled MG analogs [32]. A derivative of PP-F11N with double triazole substitution at DGlu^6^-Ala^7^ and Tyr^8^-Gly^9^ showed improved tumor targeting in A431-CCK2R xenografted athymic nude mice [28]. 

Most of the strategies directed towards stabilization of MG analogs focused on stabilizing substitutions close to the N-terminus of the peptide, since modification within the C-terminal four amino acids of the peptide sequence may potentially affect the receptor affinity [6,37]. Different metabolic stability studies revealed that cleavage sites are also present within the C-terminal receptor binding sequence -Trp-Met-Asp-Phe-NH_2_ [13,14]. Recently, it was shown that improved metabolic stability and tumor targeting is achievable by site-specific substitutions within the C-terminal receptor-binding sequence [24,38]. A small library of peptide analogs derived from DOTA-MG11 with specific amino acid substitutions within the receptor specific C-terminus, such as aromatic and N-methylated amino acids, was developed and preclinically evaluated. The targeting properties of selected derivatives are summarized in Table 3. Single substitution of the C-terminal phenylalanine with 1-naphthylalanine (1-Nal) or N-methyl-phenylalanine was investigated, as well as additional substitution of methionine with phenylglycine or N-methyl-norleucine ((N-Me)Nle) [24,38]. The most promising compound from the series was DOTA-MGS5, with replacement of Met^6^ by (N-Me)Nle and of Phe^8^ by 1-Nal. DOTA-MGS5 radiolabeled with different radiometals exhibited an enhanced cell uptake, as well as enhanced stability against enzymatic degradation and improved tumor uptake [24]. The tumor uptake of DOTA-MGS5 labeled with indium-111, gallium-68 or lutetium-177 in A431-CCK2R tumor xenografted athymic nude mice with values of 23–24%IA/g was increased by a factor of 12 when compared to [^111^In]In-DOTA-MG11. DOTA-MGS5 labeled with different trivalent radiometals also showed improved tumor uptake, as well as tumor-to-kidney ratio over [^111^In]In-CP04 (PP-F11) or [^177^Lu]Lu-PP-F11N, and is therefore most promising for clinical translation [39]. Recently the synthesis of [^68^Ga]Ga-DOTA-MGS5 using an automated synthesis module was validated and non-clinical safety studies in support of a first exploratory clinical trial were carried out for this new CCK2R targeting PET imaging agent [40]. Additional stabilization in the N-terminal part of DOTA-MGS5 by introducing the amino acid proline containing a cyclic pyrrolidine side chain in different positions led to comparable metabolically stable analogs with a biodistribution profile similar to DOTA-MGS5 [30,41]. The introduction of the above C-terminal amino acid substitutions was also investigated for CP04 in combination with additional N-methylation of Gly in position 9. Evaluation of the biodistribution profile of the ^68^Ga-labeled compounds in athymic nude mice bearing AR42J xenografts revealed a similar increase in tumor uptake, but resulted in a higher kidney retention [42].

In addition to the development of peptide analogs with improved metabolic stability and tumor targeting, alternative strategies to improve the tumor-specific uptake have been pursued. Next to receptor affinity and radioligand stability, the tumor uptake depends on the (over)expression of the specific target on the tumor cell. The ability of different kinase inhibitors to enhance the uptake of [^177^Lu]Lu-PP-F11N into CCK2R-expressing cells has been recently investigated. Pharmacological inhibition of the mTORC1 pathway using the allosteric mTORC1 inhibitor RAD001 (Everolimus) increased the level of CCK2R in AR42J and A431-CCK2R cells and consequently increased the receptor-specific cell uptake. Biodistribution studies with [^177^Lu]Lu-PP-F11N and RAD001 in the A431-CCK2R tumor mouse model revealed that only the receptor-specific tumor uptake was increased (11% IA/g versus 7%IA/g without RAD001), while the uptake in other tissues with physiological receptor expression, such as the gastrointestinal tract, remained unchanged. Combined treatment with RAD001 not only increases receptor-specific uptake of [^177^Lu]Lu-PP-F11N, but also has the potential to increase tumor radiosensitivity, as a radiosensitizing effect has been demonstrated for RAD001 in different cancer cells [43]. In this way, an overall increase of the therapeutic response in PRRT could be achieved while limiting the dose delivered to other CCK2R-expressing organs, mainly the stomach [25,43].

It is particularly important to increase the radiation dose delivered to the tumor cell when using PRRT for the treatment of minimal residual disease. Furthermore, for assessing the tumor burden and dosimetry planning prior to therapy, radionuclides with longer half-lives are needed to allow monitoring of the tumor uptake and biodistribution of the radiopeptide over several days. New radionuclides addressing these needs are emerging. Recently, the therapeutic potential of targeted alpha particle therapy with [^225^Ac]Ac-PP-F11N has been assessed in the A431-CCK2R-tumor mouse model [44]. A CCK2R-specific tumor uptake in A431-CCK2R xenografts and a dose-dependent inhibition of tumor growth has been reported, leading to an extended mean survival of treated versus untreated animals. No signs of acute radiation toxicity were observed, especially for the stomach and kidneys. However, further studies are needed to assess the long-term toxicity related to the treatment [44]. Furthermore, alternative PET and SPECT radionuclides such as copper-64 and terbium-155 are being investigated to overcome the short half-life of gallium-68 or the suboptimal image quality of indium-111. Copper-64 with a half-life 12.7 h and a β+ energy of 0.653 MeV allows for imaging at later time points and with better resolution when compared to gallium-68. In small animal PET imaging using ^64^Cu-labeled CP04 in SCID mice bearing A431-CCK2R xenografts, an up to threefold higher tumor uptake was observed over the ^111^In- and ^68^Ga-labeled compounds [45]. On the other hand, higher background levels were observed due to in-vivo instability of the ^64^Cu-chelator complex. In preclinical imaging studies on a small animal SPECT scanner, excellent imaging quality in athymic nude mice bearing A431-CCK2R xenografts was achieved using a ^155^Tb-labeled MG derivative [46]. Terbium-155 with a half-life of 5.3 days and gamma emission of 87 and 105 keV is an alternative over indium-111 for SPECT imaging and dosimetry planning.

So far, only peptide analogs with agonistic properties have been used for CCK2R targeting. However, the use of CCK2R agonists is connected to possible (mass dependent) side effects, comparable to the adverse effects after injection of pentagastrin [10]. A potential limitation of the peptide mass to be injected, could stipulate the upper limit of radioactivity which can be used for treatment and eventually result in suboptimal cancer treatment response, especially for PRRT. For high-affinity somatostatin receptor (SSTR) antagonists, a higher tumor uptake compared to that of the corresponding agonists was demonstrated. The improved tumor uptake seems to be related to the interaction with a larger number of binding sites, as no internalization of the ligand-receptor complex occurs. Using radiolabeled SSTR antagonists, an improved diagnostic efficacy was also achieved in tumors with low receptor density [47,48]. The rapid development of radiolabeled SSTR antagonists led to the development of other radiolabeled antagonistic vectors with reduced physiologic activity [33,34,35]. Nastorazepide (Z-360), a CCK2R antagonist with antiproliferative effects in gastrointestinal cancer models, has been recently evaluated in combination with gemcitabine in patients with metastatic pancreatic cancer [36]. This benzodiazepine derivative has been used for the design of the first CCK2R antagonist-based radiolabeled ligands. The initial studies focused on evaluating the spacer chemistry and different chelators for labelling of the nonpeptidic compounds with ^99m^Tc, proving the feasibility of specific tumor targeting in athymic nude mice bearing xenografts based on stably transfected HEK293 cells expressing human CCK2R or the CCK2i4svR splice variant [49]. A first head-to-head comparison of a nonpeptidic antagonist and a peptide agonist was performed using [^99m^Tc]Tc-DGA1, containing Z-360 conjugated to an acyclic tetraamine chelator through a PEG_3_-(DGlu)_4_-Lys spacer, and [^99m^Tc]Tc-Demogastrin 2, based on the gastrin analog MG0 conjugated to the same chelator via Gly [50]. [^99m^Tc]Tc-DGA1 revealed a superior metabolic stability over [^99m^Tc]Tc-Demogastrin 2 in the blood of healthy Swiss Albino mice. Consequently, the CCK2R antagonist also displayed a higher tumor uptake in SCID mice bearing HEK293-CCK2R and HEK293-CCK2i4svR xenografts. However, this was accompanied by a higher kidney retention, impairing the tumor-to-kidney ratios for both radioligands. Furthermore, DOTA-conjugated CCK2R antagonists for possible theranostic use have recently been developed [31,51]. Z-360 conjugated to DOTA through a combination of a tripeptide linker and PEG spacers of different length yielded derivatives with retained CCK2R affinity and antagonistic properties. The DOTA-conjugates radiolabeled with indium-111, lutetium-177 and gallium-68 further showed improved hydrophilicity [31,51,52]. [^111^In]In-IP-001, containing Z-360 conjugated to DOTA through a short alkyl chain followed by a PEGylated portion, has been evaluated in a human cell line with physiological CCK2R expression. Based on screening of different cancer cell lines for CCK2R expression, the authors selected A549 non-small cell lung cancer cells for the generation of xenografts in BALB/c nude mice. Small animal SPECT/CT imaging with [^111^In]In-IP-001 resulted in overall high background activity, combined with low tumor uptake. However, specificity of the tumor uptake could not be confirmed in an additional blocking experiment [51].

## 3. Tumor Imaging with Radiolabeled Gastrin Analogs

Survival of cancer patients largely depends on early localisation of the disease and accurate assessment of its extent, as well as early detection of locoregional recurrence and distant metastases. Therefore, there is a need to develop imaging diagnostic strategies tailored to the specific, possibly unique, features of the tumor. Radiolabeled gastrin analogs may be applied in the visualization of various forms of CCK2R-expressing neoplasms in order to determine the range of the surgery and positioning of locoregional and systemic therapies in a personalised approach to current diagnostic and therapeutic algorithms in these tumors.

Serum calcitonin (Ct) concentration is recognized as an accurate estimation of tumor burden in MTC patients [53,54]. Repeatedly detected Ct concentration above 10 pg/mL suggests persistence of the disease [55]. However, due to the lack of sensitive imaging tools to detect small MTC foci, basal Ct of 150 pg/mL is still a recommended threshold value for imaging procedures, as set by scientific societies. However, the wider use of PET/CT imaging with new tracers, resulting in higher sensitivity, may change this recommendation [53,55,56,57,58].

Current clinical guidelines of different scientific societies recommend the use of various radiopharmaceuticals for MTC imaging. The clinical practice guidelines of the National Comprehensive Cancer Network recommend conservative surveillance with repeated measurement of the serum markers, as well as additional imaging studies, including also [^18^F]FDG and [^68^Ga]Ga-DOTA-TATE PET/CT, in MTC patients with Ct concentrations above 150 pg/mL and negative conventional imaging [56]. The European Association of Nuclear Medicine (EANM) [59] and the European Society for Medical Oncology (ESMO) [55] also recognize [^18^F]FDOPA PET/CT as very useful in clinical practice. According to the ESMO guidelines, work-up for distant metastases with [^18^F]FDOPA PET/CT should be performed if Ct concentrations exceed 500 pg/mL, or when clinical findings are suspicious [55]. In a meta-analysis performed by Lee et al., [^18^F]FDOPA PET was found to have the highest detection rate of recurrent MTC among five PET radiopharmaceuticals: [^18^F]FDG, [^18^F]FDOPA, ^68^Ga-labeled somatostatin analogs, 3-O-methyl-6-[^18^F]fluoro-DOPA, and [^11^C]methionine [60].

Despite the promising preclinical data presented above, there are only a few clinical studies or case reports on the use of radiolabeled gastrin analogs in diagnostic imaging in MTC patients to support their efficacy. Nevertheless, based on the current clinical research, radiopharmaceuticals targeting CCK2R should be considered as sensitive and highly specific biomarkers. However, in order to obtain sufficient evidence and to establish the role of radiolabeled CCK2R analogs for imaging in the diagnostic and therapeutic algorithm in MTC patients, it is necessary to obtain more clinical data. This is hindered by the rarity of these tumors, resulting in usually small numbers of patients included in the individual studies.

A limited number of radiolabeled CCK2R-targeting peptide analogs that have been tested in pilot clinical trials in humans achieved receptor targeting, as well as safety, tolerability and, most importantly, tumor-to-background ratios sufficient for potential clinical use. A phase I clinical trial using the novel minigastrin analog [^111^In]In-CP04 for personalized diagnosis and therapy in patients with progressive or metastatic MTC (GRAN-T-MTC; ClinicalTrials.gov (accessed on 22 September 2021): NCT03246659) was conducted in the framework of the international ERA-NET on Translational Cancer Research (TRANSCAN; FP7) [19]. For the purposes of this trial, the peptide analog CP04 was selected because of its favorable pharmacokinetic properties (high metabolic stability and receptor affinity, high and persistent tumor uptake against low kidney retention) among several gastrin analogs evaluated in comparative studies performed within the COST Action BM0607 [11,12,13,61].

**Figure 2:**
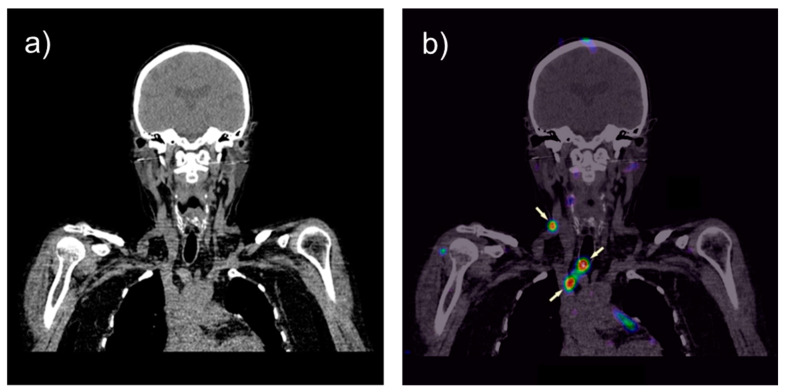
CT (**a**) and fused [^111^In]In-CP04 SPECT/CT (**b**) images in coronar orientation. Neck lymph node metastases and tumor infiltration around the tracheotomy tube (arrows) in a female patient with MTC are visible; images performed 24 h after injection of 210 MBq (50 µg peptide). Scan was performed within GRAN-T-MTC (ERA-NET on Translational Cancer Research (TRANSCAN), First Joint Transnational Call (JTC 2011) on: “Validation of biomarkers for personalised cancer medicine”, funded by the European Commission under the Seventh Framework Programme (FP7) with the following national co-found institutions: Ministry of Health (MoH), Italy, National Centre for Research and Development (NCBiR), Poland, Federal Ministry of Education and Research (BMBF), Germany, Austrian Science Fund (FWF, Project No. I1224-B19), Austria, Ministry of Higher Education, Science and Technology (MHEST), Slovenia, and General Secretariat for Research and Technology, Ministry of Education, Life Long Learning and Religious Affairs (GSRT), Greece.

The primary objectives were to determine the safety of the intravenous administration, the biodistribution and dosimetry of [^111^In]In-CP04 in cancer and normal tissues and critical organs, as well as the ability of visualization of MTC lesions. Safe use of the tested compound in humans was confirmed, although there was a marked increase in Ct and procalcitonin concentrations in the blood after tracer injection in some cases of far advanced MTC. In all patients, [^111^In]In-CP04 uptake was found in MTC lesions regardless of the peptide dose injected (10 or 50 µg), mainly in cervical and mediastinal lymph nodes and liver metastases. In two patients, detection of tumor lesions not identified in CT and MRI, was achieved (cervical lymph nodes metastases were confirmed by histopathology). Infusion of the gelatin-based plasma expander Gelofusine reduced the radiation dose to the kidneys by 53%. Pharmacokinetic data of [^111^In]In-CP04 were also used for the estimation of the radiation dose that would have been absorbed by the tumor lesions if CP04 was labeled with lutetium-177 [62,63]. In Figure 2, an example of SPECT/CT imaging using [^111^In]In-CP04 in a female patient with advanced MTC is given, showing different lesions in the neck region. The details of the final analyses of the clinical study will be published shortly.

^68^Ga-labeled CCK2R-targeting peptide probes for PET imaging in MTC will probably be increasingly used in clinical practice, however, to date the clinical data are sparse [39]. In 2016, Kunikowska et al. used a SSTR and a CCK2R targeting peptide analog, both radiolabeled with gallium-68, to visualize primary MTC in the right thyroid lobe of a male patient obtaining good tumor visulization with both radiopeptides [64]. Using the CCK2R targeting peptide, a lower uptake in liver and kidneys as compared to ^68^Ga-SSTR PET was seen, together with physiological uptake in the stomach.

**Figure 3:**
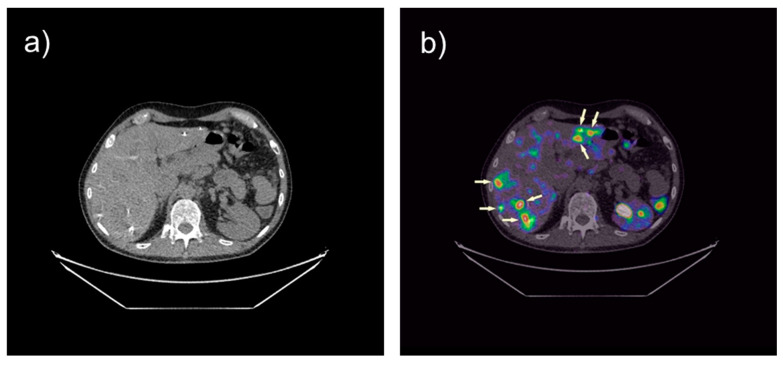
CT (**a**) and fused [^68^Ga]Ga-DOTA-MGS5 PET/CT (**b**) images in transverse orientation. Multiple liver metastasis (arrows) in a male patient with advanced MTC are visible; images performed 4 h after injection of 165 MBq (<50 µg peptide); courtesy of Prof. Alicja Hubalewska-Dydejczyk.

The first experience with the novel ^68^Ga-labeled minigastrin analog [^68^Ga]Ga-DOTA-MGS5 in comparison to [^18^F]FDOPA was described by Uprimny et al. in 2021 in a female patient with advanced MTC. PET/CT with [^68^Ga]Ga-DOTA-MGS5 vs. [^18^F]FDOPA showed less cervical lymph node and bone metastases with lower SUV values (lymph nodes 1 vs. 2, SUVmax 3.4 and 2.6 (1/2 h p.i.) vs. 11.7; bone metastasis 1 vs. 3, SUVmax 1.6 and 2.3 (1/2 h p.i.) vs. 4.0). However, [^68^Ga]Ga-DOTA-MGS5 allowed a better discrimination of liver lesions (SUVmax 6.4 and 8.3 (1/2 h p.i.) vs. 3.73), three additional metastases were found, and physiological liver activity was lower resulting in higher tumor/non-tumor ratios [65]. In Figure 3 PET/CT imaging with [^68^Ga]Ga-DOTA-MGS5 PET/CT in a male patient with advanced MTC is presented, showing several liver lesions.

Recent research on diagnostic imaging with radiolabeled CCK2R-targeting peptide analogs revealed the potential usefulness of these compounds in the management of MTC patients. CCK2R imaging may be preferable to SSTR imaging, especially in tumors with low or missing SSTR expression [3,5] and may thus be considered in the earlier stages of diagnostic schemes to optimize the procedures with a more effective strategy, allowing for radical surgery in a larger proportion of patients in order to improve survival. The accurate evaluation of the CCK2R status in individual tumor foci may be of special importance in terms of radionuclide therapy planning. Furthermore, the possibility of assessing CCK2R expression by radiolabeled MG analogs in addition to identifying the genetic profile of the tumors would help to stratify the risk of an unfavorable course of the disease with higher precision in near future. It is known that the different biological behavior of the mutations is responsible for differences in the latency period and aggressiveness of MTC [66,67,68]. Therefore, an individual genomic/proteomic MTC pattern might drive the choice of functional imaging planning with different available radiopharmaceuticals [69].

## 4. Tumor Therapy with Radiolabeled Gastrin Analogs

CCK2R is also an interesting target for PRRT. Among tumors expressing CCK2R, MTC is of special interest because of its high receptor incidence in more than 90% of MTC [1] and, on the other hand, the limited therapy options in patients with advanced stages of MTC. Standard therapy of MTC consists in total thyroidectomy and compartment-oriented lymph node dissection, which is the only chance to achieve cure. Patients with postoperative normalisation of the tumor marker calcitonin can be considered “biochemically cured” and have a favorable prognosis [55]. However, in roughly 10% of all patients, distant metastases are present already at the time of diagnosis, and are discovered in further 19–38% of patients during follow-up [55]. Furthermore, initial postoperative biochemical cure is very unlikely in patients with >10 lymph node metastases, and the chances to achieve a long-term biochemical cure are only realistic in patients with very low numbers of positive nodes [58,70]. Until several years ago, when the multikinase inhibitors cabozantinib and vandetanib were introduced as first-line systemic therapy for MTC, systemic treatment options for MTC patients were very limited, since chemotherapy has historically yielded poor results and MTC lacks uptake of radioiodine [55,58]. For tumors harbouring “rearranged during transfection” (RET) mutations, the situation recently improved further with the approval of selective inhibitors of the RET-oncogene, showing higher response rates combined with less side effects [71]. However, the systemic therapies currently approved for MTC have not been shown to improve overall survival yet, and evidence-based guidance on when to start these therapies in patients is still lacking [55]. Therefore, the need for further, effective systemic therapies remains.

MTC was chosen for the first and to date the only published treatment (phase I) study with the CCK2R agonist [^90^Y]Y-DTPA-MG0 [8]. Eight patients with advanced and rapidly progressing metastatic MTC received up to 1.85 GBq/m^2^ body surface, resulting in partial remission (*n* = 2), stable disease (*n* = 4) and progressive disease (*n* = 2) within a follow-up period of 12-36 months. Unfortunately, high hematologic and renal toxicity prevented further trials with this compound. In the course of the following years, further development resulted in MG analogs with improved biodistribution. One of these, [^177^Lu]Lu-PP-F11N, was chosen for a clinical phase 0 trial as a proof of principle study (ClinicalTrials.gov (accessed on 22 September 2021): NCT02088645) [20]. This compound showed a promising biodistribution with specific uptake in MTC. Dosimetry after infusion of 1 GBq of [^177^Lu]Lu-PP-F11N revealed radiation doses to MTC lesions sufficient for therapy, combined with low radiation doses absorbed in kidneys and bone marrow. The highest radiation doses were seen in the stomach, which would most likely be the dose-limiting organ. The administration of Gelofusine revealed no significant effect on the already low dose absorbed by the kidneys. 

**Figure 4:**
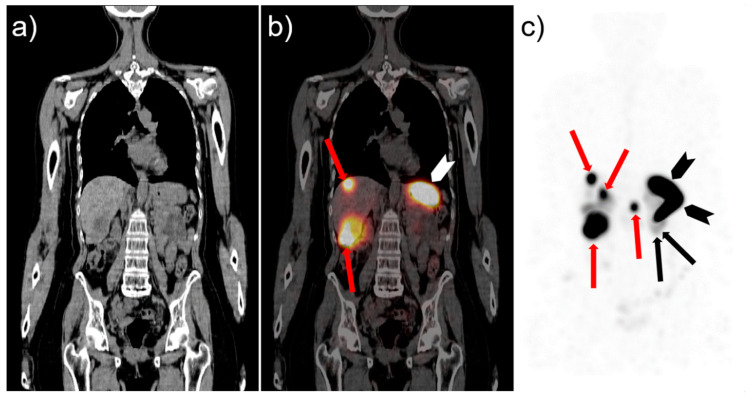
CT (**a**) and fused [^177^Lu]Lu-PP-F11N SPECT/CT (**b**) images in coronal orientation. Corresponding maximum intensity projection image of SPECT data (**c**). Images 72 h after infusion of 6.3 GBq (<100 µg peptide) in a female patient with hepatic MTC metastases demonstrates strong uptake of the radiopharmaceutical in hepatic metastases and stomach. Red arrows: Liver metastases; white and black arrowheads: stomach; black arrows: Kidney; courtesy of Dr. Christof Rottenburger.

The acute toxicity of [^177^Lu]Lu-PP-F11N was low with self-limiting adverse events not higher than grade 1, according to Common Terminology Criteria for Adverse Events version 4.03 (CTCAE). Based on the dosimetric calculations, it was stated that fractionated therapy with 50 GBq [^177^Lu]Lu-PP-F11N should be possible without surpassing maximum tolerated doses of risk organs. In consequence, a phase 1 dose escalation study was initiated (ClinicalTrials.gov (accessed on 22 September 2021): NCT02088645). Within this trial, the first planned escalation cohort (n = 3) received 3 × 6 GBq without dose limiting toxicity or other toxicity higher than grade 2, according to CTCAE [72]. In all patients, at least a temporary reduction of tumor markers occurred after therapy. In Figure 4, an exemplary SPECT/CT image of a patient with hepatic MTC metastases is given, showing specific uptake of [^177^Lu]Lu-PP-F11N in the liver lesions together with physiological uptake in the stomach. Therefore, the study continued with the next dose escalation step (4 × 8 GBq).

As the clinical evaluation of CCK2R-targeted therapies has only started recently and to date has achieved only preliminary results, this approach is yet to be mentioned in any guidelines for the treatment of MTC. The American Thyroid Association Guidelines recommend the consideration of two approaches of therapy with radiolabeled tracers in selected patients with MTC: SSTR-targeted PRRT and pretargeted anti-CEA radioimmunotherapy [58]. Both strategies could be interesting alternatives of targeted radiotherapy, especially in patients with poor CCK2R expression of tumors [73,74].

## 5. Perspectives

In recent years, the number of novel target-specific radiotracers introduced to clinical practice has been growing rapidly. This is not only due to the introduction of novel tumor targeting molecules but also due to the increasing availability of radionuclides, both for diagnostic imaging in PET and SPECT and for PRRT [75,76]. The overexpression of CCK2R on MTC cells and other tumors provides attractive perspectives for imaging and targeted therapy with radiopharmaceuticals.

As summarized in this review, the drawbacks of the first gastrin analogs developed, related to high kidney uptake or low enzymatic stability, were overcome by specific modification of the amino acid sequence. Today, new peptide analogs with improved targeting profiles are available which are mature for patient use. Although the process of their clinical validation is still in its infancy, the new optimized peptide analogs promise to make an important contribution to the diagnostic work-up and treatment of patients with CCK2R-expressing malignancies. A summary of the status of recent clinical trials is given in Table 4. 

Further aspects that should be addressed in the future to advance the clinical application of CCK2R targeting peptides include the continuation of tracer design as well as the initiation of new clinical trials investigating the efficacy of CCK2R-targeted PRRT.

Besides the co-injection of enzyme inhibitors to overcome rapid enzymatic degradation of the radiolabeled peptide agonists, the development of nonpeptidic CCK2R antagonists is another strategy that has recently been pursued to further enhance pharmacokinetics and tumor targeting. The field of radiolabeled CCK2R antagonists is just emerging, and further studies are needed to investigate whether, similar to the somatostatin/SSTR system, CCK2R antagonists have the potential to outperform the agonists. Other structural modifications of peptide agonists, alternative to those described in this review, also have potential for further improvement. The biological behavior of gastrin analogs might require radionuclides with longer half-lives than that of gallium-68 to allow proper visualization of the tumor sites [64]. The use of positron emitters with longer half-lives not only is expected to improve diagnostic imaging, but also may allow for improved dosimetry calculation for therapy. Scandium and copper radioisotopes are particularly interesting in this respect, as they can provide true theranostic pairs, allowing the use of radioactive isotopes of the same element for imaging (scandium-43/44, copper-64) and therapy (scandium-47, copper-67) [76,77]. In addition, radionuclides with improved physical properties for therapy are being investigated. Terbium-161, next to beta minus particle and gamma ray emission, also emits Auger/conversion electrons, with the potential to enhance the therapeutic effect [46,78]. Therapy with alpha emitters holds the promise of effective tumor treatment. Thus, besides actinium-225 and bismuth-213, different new alpha emitters are in the focus of the development. Further progress in the production process is needed to advance in the availability of these new emerging radionuclides for clinical use [76]. Finally, the choice of the labelling method also plays an important role for the overall performance of the radiopharmaceutical. For the labelling with trivalent radiometals, bifunctional chelators other than DOTA could be considered as an attempt to improve the pharmacokinetic profile [79].

The clinical data presented in this review emphasize the potential value of radiolabeled MG analogs in the diagnostic work-up of CCK2R expressing neoplasms. However, the possible integration of CCK2R-targeted PRRT in the therapy algorithm of MTC is not clear yet, and will depend on the results of ongoing and future clinical trials. The main indication of this new treatment strategy might be metastasized MTC. In this scenario, sufficient CCKR2 expression of the tumor needs to be confirmed prior to PRRT via diagnostic CCK2R imaging (PET/CT) in a theranostic approach, comparable to the established procedure in SSTR-targeted PRRT. In view of the recent, promising developments in systemic oncologic treatment, CCK2R-targeted PRRT might be of particular interest for patients lacking RET mutation and with resistance to RET and/or other multikinase inhibitor treatment, respectively, with unbearable side effects or with contraindications to such therapies. Furthermore, the evaluation of possible combinations of kinase inhibitors and CCK2R-targeted PRRT, as well as the prospective comparison of both approaches would be of interest in future clinical trials. Synergistic effects might be possible, as e.g., upregulation of CCK2R with consecutively improved tumor uptake after pre-treatment with the mTOR inhibitor Everolimus was reported in a preclinical study [43], as mentioned above. Another promising approach might be the application of CCK2R targeted therapy in an adjuvant setting, in a comparable way to the use of radioiodine in the treatment of differentiated thyroid carcinoma after total thyroidectomy. A single cycle of CCK2R-targeted PRRT, e.g., in high risk patients after thyroidectomy and neck dissection, might improve the chances of achieving biochemical remission and allow the assessment of metastatic disease by post-therapy scintigraphy. A further perspective is the use of radionuclides with properties adapted to the aimed application, e.g., the use of radionuclides with high linear energy transfer, such as Auger-electron or alpha emitters, when the therapy targets predominantly small volume lesions, as to be expected in an adjuvant approach.

## 6. Conclusions

The overexpression of the CCK2R in MTC and other tumors provides attractive perspectives for imaging and targeted therapy with radiopharmaceuticals. Patients with MTC are lacking sensitive imaging modalities, allowing the detection of small metastases as well as effective treatment strategies at an advanced stage. The role of nuclear medicine in these unmet clinical needs is yet to be demonstrated. Different CCKR targeting tracers are the subject of preclinical and clinical investigation. Based on the current state of radiopharmaceutical development and the clinical translation, three new CCK2R peptide agonists, [^111^In]In-CP04, [^68^Ga]Ga-DOTA-MGS5 and [^177^Lu]Lu-PP-F11N, are currently available for clinical application in SPECT, PET and PRRT within clinical studies. In first patient studies, the new radiopharmaceuticals showed the potential to visualize additional tumor lesions not detectable by conventional CT and MRI. The ^68^Ga-labeled tracer is of particular interest for clinical practice and allows for a better discrimination of hepatic lesions due to very low physiological liver uptake. Based on the low toxicity profile of initial PRRT in three patients, the new MG analogs with improved metabolic stability and reduced kidney uptake promise to have a significant impact in the diagnosis and therapy of CCK2R related malignancies. Further preclinical development and clinical evidence will be reported in the near future to support these initial achievements.

## Figures and Tables

**Table 1:** Tumor targeting properties of selected radiolabeled CCK2R targeting peptide analogs.

CCK2R Targeting Peptide	Radiopeptide Injected	Tumor Xenograft/ Mouse Model	Tumor Uptake ^1^	Tumor Uptake ^1^ + PA Co-Injection ^2^	Ref.
DOTA-DGlu-Ala-Tyr-Gly-Trp-Met-Asp-Phe-NH_2_	[^111^In]In-DOTA-MG11	AR42J/SCID A431-CCK2R/SCID	<2% IA/g 2.5% IA/g	15% IA/g 16% IA/g	[21]
DOTA-DGlu-(Glu)_5_-Ala-Tyr-Gly-Trp-Met-Asp-Phe-NH_2_	[^111^In]In-DOTA-MG0	A431-CCK2R/SCID	12% IA/g	17% IA/g	[22]
DOTA-DGlu-(DGlu)_5_-Ala-Tyr-Gly-Trp-Met-Asp-Phe-NH_2_	[^111^In]In-CP04 (PP-F11, MG48)	A431-CCK2R/SCID	9% IA/g	16% IA/g	[22]
DOTA-DGlu-Ala-Tyr-Gly-Trp-Met-Asp-Phe-NH_2_	[^177^Lu]Lu-DOTA-MG11	A431-CCK2R/athymic nude	1.5% IA/g	7% IA/g	[23]
DOTA-DGlu-(DGlu)_5_-Ala-Tyr-Gly-Trp-Nle-Asp-Phe-NH_2_	[^177^Lu]Lu-PP-F11N	A431-CCK2R/athymic nude	7% IA/g	9% IA/g	[23]
DOTA-DGlu-Ala-Tyr-Gly-Trp-(N-Me)Nle-Asp-1-Nal-NH_2_	[^111^In]In/[^177^Lu]Lu/[^68^Ga]Ga-DOTA-MGS5	A431-CCK2R/athymic nude	23–24% IA/g	Not determined	[24]

^1^ Time point post injection: 4 h p.i. for indium-111 and lutetium-177; 1 h p.i. for gallium-68; ^2^ PA = 300 µg phosphoramidon.

**Table 2:** Targeting properties of selected radiolabeled MG analogs with amide-to-triazole substitutions.

CCK2R Targeting Peptide	Radiopeptide Injected	Cell Internalization 4 h Incubation ^1^	In Vitro Stability ^2^	Tumor Uptake 4 h p.i. ^1^	Ref.
DOTA-DGlu-Ala-Tyr-Ψ[Tz]-Gly-Trp-Nle-Asp-Phe-NH_2_	[^177^Lu]Lu-TZMG **6**	>50%	<10%	3.9% IA/g	[27,29]
DOTA-DGlu-Ψ[Tz]-Ala-Tyr-Ψ[Tz]-Gly-Trp-Nle-Asp-Phe-NH_2_	[^177^Lu]Lu-TZMG **86**	>50%	>30%	6.0% IA/g	[29]
DOTA-DGlu-Ψ[Tz]-Ala-Ψ[Tz]-Tyr-Ψ[Tz]-Gly-Trp-Nle-Asp-Phe-NH_2_	[^177^Lu]Lu-TZMG **876**	~50%	~10%	6.0% IA/g	[29]
DOTA-(DGlu)_6_-Ala-Tyr-Ψ[Tz]-Gly-Trp-Nle-Asp-Phe-NH_2_	[^177^Lu]Lu-NMG **2**	>70%	>95%	7.2% IA/g	[28]
DOTA-DGlu-Ψ[Tz]-Ala-Tyr-Ψ[Tz]-Gly-Trp-Nle-Asp-Phe-NH_2_	[^177^Lu]Lu-NMG **3**	>70%	>95%	6.9% IA/g	[28]

^1^ A431-CCK2R cells were used for cell uptake studies and induction of tumor xenografts in athymic nude mice. ^2^ Intact compound 24 h after incubation in blood plasma.

**Table 3:** Targeting properties of selected radiolabeled MG analogs with substitutions within the C-terminal sequence.

CCK2R Targeting Peptide	Radiopeptide Injected	Cell Internalization 2 h Incubation ^1^	In Vitro Stability ^2^	Tumor Uptake ^1^	Ref.
DOTA-DGlu-Ala-Tyr-Gly-Trp-Nle-Asp-1-Nal-NH_2_	[^111^In]In-MGS1	~25%	~60%	1.2% IA/g	[38]
DOTA-DGlu-Ala-Tyr-Gly-Trp-(N-Me)Nle-Asp-(N-Me)Phe -NH_2_	[^111^In]In-MGS4	~25%	>95%	10% IA/g	[38]
DOTA-DGlu-Ala-Tyr-Gly-Trp-(N-Me)Nle-Asp-1-Nal-NH_2_	[^111^In]In/[^177^Lu]Lu/[^68^Ga]Ga-DOTA-MGS5	~50%	>95%	23–24% IA/g	[24]

^1^ A431-CCK2R cells were used for cell uptake studies and induction of tumor xenografts in athymic nude mice. ^2^ Intact compound after incubation in human serum for 4 h (gallium-68) or 24 h (indium-111 and lutetium-177).

**Table 4:** Summary of recent clinical studies with radiolabeled CCK2R targeting peptide analogs.

Study Acronym	Sponsor	Intervention/Treatment	Identifier	Recruitment Status
GRAN-T-MTC	Azienda Ospedaliero-Universitaria Pisana, Pisa, Italy	[^111^In]In-CP04, Gelofusine	ClinicalTrials.gov (accessed on 22 September 2021): NCT03246659	Completed
Ga-68-CCK2R PET/CT in NET	Medical University of Innsbruck, Austria	[^68^Ga]Ga-DOTA-MGS5	EudraCT: 2020-003932-26	Recruiting
Lumed phase 0/A and phase I	University Hospital Basel, Switzerland	[^177^Lu]Lu-PP-F11N, Gelofusine (phase 0/A)	ClinicalTrials.gov (accessed on 22 September 2021): NCT02088645	Completed (phase 0/A) Recruiting (phase I)
Lumed phase 0/B	University Hospital Basel, Switzerland	[^177^Lu]Lu-PP-F11N, Sacuitril	ClinicalTrials.gov (accessed on 22 September 2021): NCT03647657	Recruiting
